# Naringenin Improves Ovalbumin-Induced Allergic Asthma in Rats through Antioxidant and Anti-Inflammatory Effects

**DOI:** 10.1155/2022/9110798

**Published:** 2022-04-04

**Authors:** Seyed Vahid Jasemi, Hosna Khazaei, Sajad Fakhri, Ehsan Mohammadi-Noori, Mohammad Hosein Farzaei

**Affiliations:** ^1^Department of Internal Medicine, Faculty of Medicine, Kermanshah University of Medical Sciences, Kermanshah, Iran; ^2^Medical Technology Research Center, Health Technology Institute, Kermanshah University of Medical Sciences, Kermanshah, Iran; ^3^Pharmaceutical Sciences Research Center, Health Institute, Kermanshah University of Medical Sciences, Kermanshah, Iran

## Abstract

Asthma is a chronic disease with eosinophilic inflammation and oxidative damages leading to airway obstruction. Naringenin is a phytochemical possessing strong antioxidant and anti-inflammatory activities against chronic destructive conditions. The current study is devoted to evaluating naringenin's effects on the attenuation of inflammation and oxidative stress in lung tissue in a rat model of ovalbumin-induced asthma. Male Wistar rats were allocated to five groups of six: normal control (NC, receiving 1 ml/day of normal saline, orally), asthmatic (AS, receiving ovalbumin (1 mg/mL), and alum (1 mg/mL in saline) on days 0 and 14. Then, on days 21, 22, and 23, they were sensitized with the inhalation of ovalbumin), AS treated with dexamethasone (AS, 1 mg/kg/day, orally) [AS + D1], AS treated with naringenin (20 mg/kg/day, orally) [AS + N20], and AS treated with naringenin (40 mg/kg/day, orally) [AS + N40]. All the groups received associated drugs/agents for 28 days. Finally, bronchoalveolar lavage fluid (BALF) and lung tissue samples were taken off from the animals. The eosinophil count in BALF and malondialdehyde (MDA), glutathione (GSH), interleukin-13 and -4 (IL-13 and IL-4) levels were measured. Besides, the expression of urocortin (UCN) and surfactant protein-D (SP-D) were evaluated in the lung tissue using immunohistochemistry (IHC) and western blotting methods, respectively. Hematoxylin and eosin (H&E) staining were utilized to conduct histopathological analysis. Naringenin treatment significantly reduced MDA, remarkably increased GSH, and meaningfully reduced IL-4 and IL-13 levels in lung tissue. The count of eosinophils in the BALF of AS + N20 and AS + N40 was significantly reduced in comparison with the AS group. The UCN and SP-D protein levels were significantly decreased in the AS + N20 and AS + N40 groups compared to the AS group, using the IHC and western blot methods, respectively. Histopathological analysis data also confirm the results. Naringenin improves the symptoms of allergic asthma through antioxidant and anti-inflammatory effects.

## 1. Introduction 

Asthma is an airway-associated complicated disease, diagnosed by reversible obstruction/stenosis/restructuring, severe inflammation, and oxidative stress. Such a condition is followed by structural or tissue alterations that occur at all levels of the bronchial respiratory tract and affect the whole layers of the respiratory tract [1]. These changes are included with increased smooth muscle mass due to cell hyperplasia and hypertrophy, epithelial surface metaplasia, goblet cell hyperplasia, increased mucus secretion, elevated number and diameter of vessels, and subepithelial fibrosis [2]. Airway inflammation is a complex molecular process that begins with an increase in inflammatory mediators and has now been shown to occur in response to increased oxidative stress [3]. During the inflammation process, migration of inflammatory cells to the mucosa and epithelium, especially those of eosinophils and mast cells, will be increased in the mucosa and the bronchoalveolar lavage fluid (BALF) of the respiratory tract [4]. In addition to the inflammatory nature of asthma, the role of oxidative stress in the pathogenesis of such a disease is also highlighted. It has been shown that serum antioxidant capacity is dramatically decreased during persistent asthma and asthma attacks, while oxidative mediators are increased [5–7]. An imbalance in the levels of oxidative mediators and inflammatory pathways restricts airflow and increases airway sensitivity. In this line, the lack of anti-oxidants and anti-inflammatory agents in the airways of patients with asthma exacerbates/increases the inflammatory response in asthmatic patients [8, 9].

From the therapeutic point of asthma, prevailing studies have shown the severe side effects of long-term use of asthma medications (e.g., corticosteroids), damaging the kidney, liver, heart, digestive system, immune system, and emergence of types of cancer [10, 11]. It urges the need for finding novel alternative treatments to combat asthma complications, which possess higher efficacy and lower side effects. The plant kingdom, especially flavonoid, has shown a wide range of antioxidant and immunomodulatory activities with the potential to fight asthma [12–14].

Naringenin (4, 5, 7-trihydroxyflavanone) is a flavonoid derived from grapes and citrus, which has recently shown positive effects on the respiratory system [15]. This flavonoid has also been proven to possess various pharmacological properties such as anticancer, antimutation, and antiatherogenic [16, 17]. Naringenin is found in citrus fruits, tomatoes, and a species of fig called *Ficus carica* [18]. The anti-inflammatory properties of orange juice were exerted by inhibiting the production of nitric oxide and prostaglandin E2. Besides, naringenin's antioxidant and hypoglycemic properties have been confirmed by reducing oxidative mediators [19–25]. In this regard, previous studies have shown the effectiveness of naringenin in treating airway inflammatory diseases such as chronic obstructive pulmonary disease, asthma, and coronavirus disease 2019 [26–28]. Studies have shown that naringenin can improve chronic inflammation and remodelling of lung tissue through anti-inflammatory mechanisms, including reducing T helper type 2 (Th2) cytokines and decreasing immunoglobulin E levels in BALF [29]. Also, it has been proven that naringenin chalcone improves inflammation in allergic asthma by inhibiting cluster of differentiation 4T (CD4T) cells activity and the production of histamine from mast cells. It also reduces the production of cytokines, including interleukin (IL)-4, IL-5, and IL-13, and reduces mucus secretion [30]. It is well known that IL-13 causes an over-reaction in the airways and IL-4 stimulates mucus secretion, which are obvious indicators of asthma [31]. Naringenin has also been shown to inhibit the expression of thymic stromal lymphopoietin, an important marker of allergic disease, and also reduce nuclear factor kappa-light-chain-enhancer of activated B cells (NF-*κ*B) activity [32]. The anti-inflammatory mechanism effect of naringenin has been shown in additional studies to work by inhibiting the NF-*κ*B and mitogen-activated protein kinase signal pathways. Such an effect was applied by reducing the levels of inflammatory cytokines such as IL-33, tumor necrosis factor alpha (TNF-*α*) and IL-1*β* that are associated with reducing the symptoms of inflammation and controlling inflammatory diseases. Hence, this phytochemical has been introduced as an immunomodulator [33].

Since chemical treatments for asthma have many side effects, low effectiveness, and predominantly play palliative roles, natural compounds such as naringenin can control the inflammatory disease by targeting the major dysregulated pathways during disease, i.e., suppressing oxidative stress and inflammation, thereby being used as an adjunct drug in the treatment of asthma. Therefore, evaluating the naringenin's therapeutic effect in the ovalbumin-induced rat model of asthma was investigated in the present work.

## 2. Materials and Methods

### 2.1. Animals

Overall, 30 male Wistar rats weighing 165–175 g were supplied from the Pasteur Institute (Tehran, Iran). The animals were exposed to controlled conditions at 22–25°C and 12 h of dark/light cycle. Rats had free access to water and food at all stages of the experiment. All stages of working with animals were approved by the ethics committee for working with animals of Kermanshah University of Medical Sciences, Iran (IR.KUMS.REC.1398.1034). The rats were divided into five groups of 6 (*n* = 6) as follows: (1) the normal control group (NC, receiving 1 ml/day normal saline, orally), (2) the asthmatic group sensitized with ovalbumin (receiving ovalbumin (1 mg/mL) and alum (1 mg/mL in saline) on days 0 and 14. Then, on days 21, 22, and 23, they were sensitized with the inhalation of ovalbumin using a nebulizer) (AS), (3) the AS group received dexamethasone 1 mg/kg by gavage (AS + D1), (4) the  AS group received naringenin (Sigma-Aldrich) 20 mg/kg/day for 28 connective days by gavage (AS + N20), and (5) the  AS group received 40 mg/kg/day naringenin by gavage (AS + N40) [34, 35]. All the groups received associated drugs/agents for 28 days.

### 2.2. Asthma Induction Protocol

The induction of asthma in animals was performed according to Moura et al. and Yang et al. methods [36, 37]. In brief, all of the groups (except NC) were sensitized with ovalbumin (Sigma-Aldrich), so the rats were intraperitoneally injected with ovalbumin (1 mg/mL) and alum (1 mg/mL in saline) on days 0 and 14. Then, on days 21, 22, and 23, they were sensitized with the inhalation of ovalbumin using a nebulizer (OMRON, NE-C29-E). Notably, group I was exempted from this stage, and they only received an injection and an inhalation of saline. Finally, a mixture of ketamine (100 mg/kg) and xylazine (10 mg/kg) was intraperitoneally injected into rats, and they were euthanized 24 h after the final dose of treatment [38]. Moreover, lung tissue and BALF samples were collected for further examinations.

### 2.3. BALF Collection

The airway lumina obtained from tracheae cannulation was washed with 6 mL of saline solution followed by centrifugation at a relative centrifugal force (RCF) of 400*G* for 10 min. The cell pellet was vortexed and suspended in a 500 mL saline solution again and used for measuring eosinophil count.

### 2.4. Tissue Preparation

The exact 1 g of lung tissue was homogenized with 2 mL of phosphate-buffered saline (pH: 7.4) and centrifuged at RCF of 10,000*G* for 15 min. The supernatant was collected to measure IL-13, IL-4, malondialdehyde (MDA), and glutathione (GSH) levels.

### 2.5. Measurement of IL-13 and IL-4

Enzyme-linked immunosorbent assays (ELISA) were conducted using specific rat IL-13 and IL-4 ELISA kits and manufacturer's directions (RayBiotech, Rat ELISA Kits, USA).

### 2.6. Measurement of MDA and GSH

The activity of GSH and MDA enzymes in lung tissue was measured using the enzymatic colorimetric method of American Cayman Chemical Company kits.

### 2.7. Immunohistochemistry (IHC) Method

To evaluate the expression of urocortin (UCN) in lung tissue, we used the IHC method. For this purpose, after fixing the samples in 4% paraformaldehyde, the tissues were immersed in 20% sucrose and then cut into 10 *μ*m pieces. The preliminary incubation process of slides was carried out by using anti-CD3 antibody (145-2C11) (eBioscience, California, USA) with a 1 : 100 dilution ratio, and the secondary and final incubation was performed by utilizing biotinylated mouse anti-hamster IgG antibody (Proteintech Group, New York, USA). The peroxidase-conjugated streptavidin system was used to visualize the reaction product, along with using 3, 3-diaminobenzidine (Serva, Heidelberg, Germany) as the substrate.

### 2.8. Western Blot Analysis

We used the western blot method to examine the expression of surfactant protein-D (SP-D) and glyceraldehyde 3-phosphate dehydrogenase (GAPDH) in the lung tissue. For this purpose, after homogenizing tissues by using RIPA lysis buffer (Upstate Biotechnology, Lake Placid, NY, USA), the sample was dissolved in a buffer containing 2 mM DTT (dithiothreitol), 12.5 mM Tris-HCl buffer (pH 6.8), 4% sodium dodecyl sulfate, 20% glycerol, and 0.004% bromophenol blue. Then, the samples were heated to 70°C and kept at that temperature for 10 min. After that, 13% polyacrylamide gels were used to electrophorese the samples. The samples were transferred to polyvinylidene difluoride (PVDF; Atto, Tokyo, Japan) membranes. The efficiency of the transfer was evaluated by staining the samples with 0.1% Coomassie blue. Blotting buffer containing 5% nonfat dry milk in PBS-0.05% Tween 20 was utilized to block unstained membranes. The blocked unstained membranes were incubated using anti-SP-D and anti- GAPDH antibodies with a dilution ratio of 1 : 2000 in a blotting buffer for two days. To pursue the experimental procedure, the membranes were washed in a blotting buffer and then incubated (60 min) with a dilution ratio of 1 : 2000 containing mouse anti-rabbit immunoglobulin G conjugated to horseradish peroxidase in a blotting buffer. The samples were rewashed with PBS-Tween solution, and finally, the enhanced chemiluminescence technique (Amersham, Piscataway, USA) detected the signals. The scanned and pixel intensity of the stained bands were quantified utilizing Scion Image 1.59 (Scion Corp., Frederick, USA) based on NIH Image [39].

### 2.9. Histopathological Analysis

Lung tissues were subjected to formalin after sampling. Then, they were dipped in a paraffin solution, cut into 5 *μ*m thick pieces, and stained using hematoxylin and eosin (H&E). Finally, they were monitored and photographed by a compound binocular light microscope [40].

### 2.10. Statistical Analysis

The data analysis was carried out by utilizing GraphPad Prism software (version 8), and the results were introduced as mean + SEM. We used the ANOVA test to evaluate significant differences between groups. Bonferroni post hoc test analysis was used, and *P* < 0.05 was considered as a significant level.

## 3. Results

### 3.1. The Effect of Naringenin on the Bodyweight of Rats

**Figure 1:**
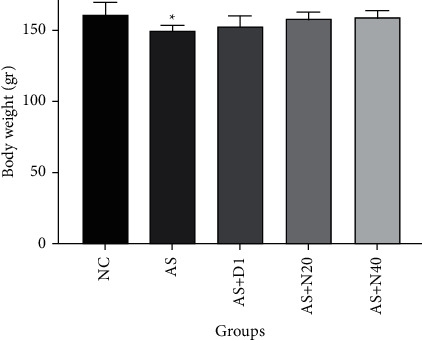
The effect of naringenin on the bodyweight. The data are expressed as the mean + SEM. ^*∗*^*P* < 0.05, compared to the NC group. NC: normal control group, AS: asthmatic group, AS + D1: the asthmatic group treated with dexamethasone 1 mg/kg/day orally, AS + N20: the asthmatic group treated with naringenin 20 mg/kg/day orally, and AS + N40: the asthmatic group treated with naringenin 40 mg/kg/day orally (*N* = 6).

According to the results obtained from statistical analysis, no significant difference was observed in the bodyweight of different groups, except for the AS group, which had a significant decrement compared to the NC group (*P* < 0.05) (Figure 1).

### 3.2. The Effect of Naringenin on the IL-4 and IL-13 in the Lung Tissue

A significant increase in the IL-4 and IL-13 levels of the AS group results was observed compared to the NC group (^*∗∗∗*^*P* < 0.001). Also, a significant increase in the AS + N20 group was observed compared to NC (*P* < 0.001). Comparing to the AS + D1 and AS + N40 groups, the AS group showed a significant decrease in IL-4 levels (*P* < 0.05 and *P* < 0.01). Besides, such reduction was observed for IL-13 levels in all treated groups, including AS + D1, AS + N20, and AS + N40 (*P* < 0.001).

**Figure 2:**
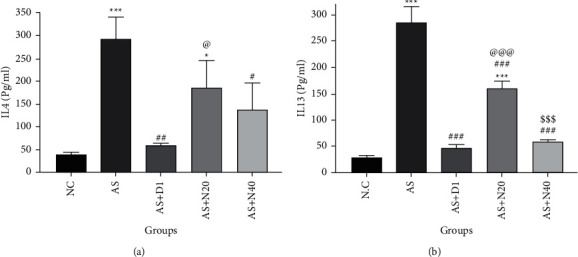
The effect of naringenin on the IL-4 (a) and IL-13 (b) in the lung tissue. The data are expressed as the mean + SEM. ^*∗*^*P* < 0.05 and ^*∗∗∗*^*P* < 0.001 compared to the NC group. ^#^*P* < 0.05, ^##^*P* < 0.01, ^###^*P* < 0.001 compared to the AS group, ^@^*P* < 0.05, ^@@@^*P* < 0.001 compared to the AS + D1 group, and ^sss^*P* < 0.001 differences between the AS + N20 and AS + N40 groups. NC: normal control group, AS: asthmatic group, AS + D1: asthmatic group treated with dexamethasone 1 mg/kg/day orally, AS + N20: asthmatic group treated with naringenin 20 mg/kg/day orally, and AS + N40: asthmatic group treated with naringenin 40 mg/kg/day orally (*N* = 6).

The obtained results from the statistical analysis confirmed that the anti-inflammatory effect of naringenin at 40 mg/kg was greater than that of naringenin at 20 mg/kg, which was affirmed by a considerable decrement in IL-13 levels in the AS + N40 group compared to the AS + N20 group (*P* < 0.001) (Figure 2).

### 3.3. The Effect of Naringenin on GSH and MDA Levels in the Lung Tissue

All groups had a significant increment in MDA levels in comparison to the NC group (*P* < 0.01 and *P* < 0.001) in lung tissue. This increment was greater in the AS group in comparison with other groups and was somehow controlled in the AS + D1, AS + N20, and AS + N40 groups. In addition, by comparing all of the groups to the AS group, a significant decrease was observed in MDA levels (*P* < 0.001 and *P* < 0.01). The AS + N40 group displayed a significant reduction in MDA levels when measured against the AS + N20 group (*P* < 0.01).

**Figure 3:**
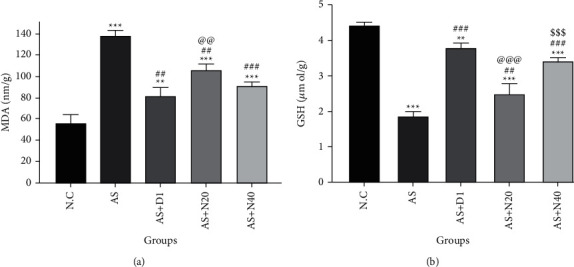
The effect of naringenin on MDA (a) and GSH (b) levels in the lung tissue. The data are expressed as the mean + SEM. ^*∗∗*^*P* < 0.01and ^*∗∗∗*^*P* < 0.001 compared to the NC group. ^##^*P* < 0.01, ^###^*P* < 0.001 compared to the AS group, ^@@^*P* < 0.01, ^@@@^*P* < 0.001 compared to the AS + D1 group, and ^sss^*P* < 0.001 differences between the AS + N20 and AS + N40 groups. NC: normal control group, AS: asthmatic group, AS + D1: the asthmatic group treated with dexamethasone 1 mg/kg/day orally, AS + N20: the asthmatic group treated with naringenin 20 mg/kg/day orally, and AS + N40: the asthmatic group treated with naringenin 40 mg/kg/day orally, (*N* = 6).

All groups experienced a significant decrement in GSH levels compared to the NC group (*P* < 0.001 and *P* < 0.01), but there was a significant increment in GSH levels between the AS + N20, AS + N40, and AS + D1 groups compared to the AS group (*P* < 0.001 and *P* < 0.01). Also, a significant increment in the AS + N40 group GSH level in comparison to the AS + N20 group confirmed the high antioxidant activity of naringenin at high doses (*P* < 0.01) (Figure 3).

### 3.4. The Effect of Naringenin on the UCN Expression in the Lung Tissue

**Figure 4:**
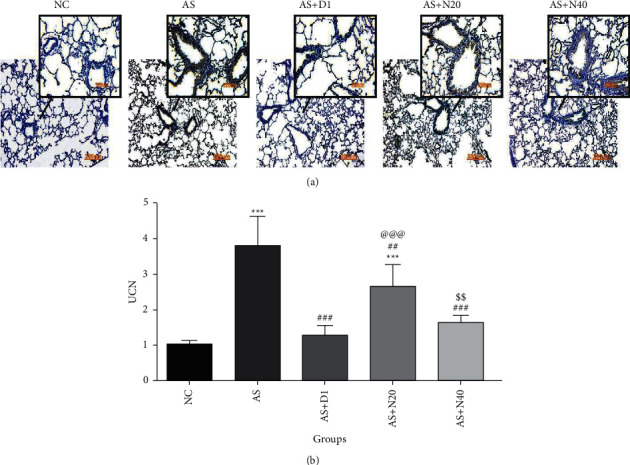
The effect of naringenin on the UCN expression in the lung tissue. (a) Immunohistochemistry images; (b) results of statistical analysis. The data are expressed as the mean + SEM. ^*∗∗∗*^*P* < 0.001 compared to the NC group. ^##^*P* < 0.01, ^###^*P* < 0.001 compared to the AS group, ^@@@^*P* < 0.001 compared to the AS + D1 group, and ^ss^*P* < 0.01 differences between the AS + N20 and AS + N40 groups. NC: normal control group, AS: asthmatic group, AS + D1: asthmatic group treated with dexamethasone 1 mg/kg/day orally, AS + N20: asthmatic group treated with naringenin 20 mg/kg/day orally, and AS + N40: asthmatic group treated with naringenin 40 mg/kg/day orally (*N* = 6).

As shown in Figure 4, UCN expression was increased in all of the groups compared to the NC group. This increment was significant in the AS and AS + N20 groups compared to the NC group (*P* < 0.001). Also, all treatment groups, including AS + D1, AS + N20, and AS + N40, showed a significant decrease in UCN expression when they were evaluated compared to the AS group (*P* < 0.01 and *P* < 0.001). Furthermore, a significant decrement was realized by comparing the AS + N40 group to the AS + N20 group (*P* < 0.01).

### 3.5. The Effect of Naringenin on Inflammatory Cells and Airway Wall Thickness in Histopathological Analysis

Considering the NC group as the reference, the airway wall thickness increased in all of the groups, which was significant in the case of the AS and AS + N20 groups (*P* < 0.01 and *P* < 0.001). In all treated groups, including AS + D1, AS + N20, and AS + N40, airway wall thickness was dramatically reduced compared to the AS group (*P* < 0.01 and *P* < 0.001).

**Figure 5:**
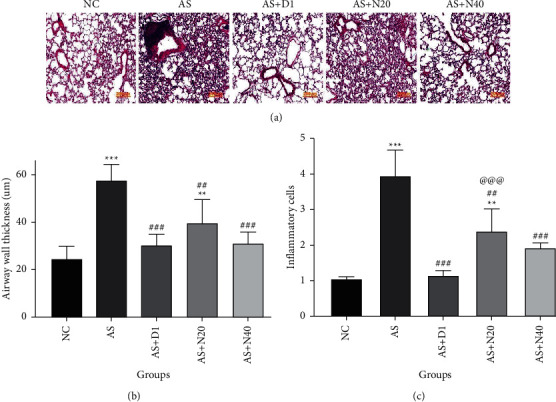
The effect of naringenin on inflammatory cells and airway wall thickness in histopathological analysis. (a) H&E staining, (b) airway wall thickness (*μ*m), and (c) inflammatory cells (fold change). The data are expressed as the mean + SEM. ^*∗∗*^*P* < 0.01 and ^*∗∗∗*^*P* < 0.001 compared to the normal control group. ^##^*P* < 0.01, ^###^*P* < 0.001 compared to asthmatic group, and ^@@@^*P* < 0.001 compared to asthmatic treated with dexamethasone.

An increment in the inflammatory cells was observed in all groups compared to the NC group, which was acutely significant in the AS and AS + N20 groups compared to the NC group (*P* < 0.01 and *P* < 0.001). When all of the treated groups, including AS + D1, AS + N20, and AS + N40, were compared to the AS group, a significant decrement was observed in the results (*P* < 0.01 and *P* < 0.001) (Figure 5).

### 3.6. The Effect of Naringenin on SP-D/GAPDH Expression in the Lung Tissue

**Figure 6:**
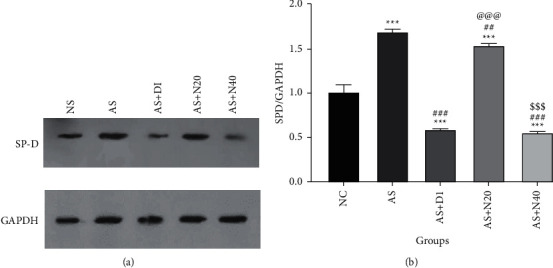
The effect of naringenin on SP-D/GAPDH expression in the lung tissue. (a) Western blotting images of SP-D expression and GAPDH expression. (b) Results of SP-D/GAPDH ratio statistical analysis. The data are expressed as the mean + SEM. ^*∗∗∗*^*P* < 0.001 compared to the normal control group. ^###^*P* < 0.001 compared to the asthmatic group, ^@@@^*P* < 0.001 compared to asthmatic treated with dexamethasone, and ^sss^*P* < 0.001 differences between the N20 and N40 groups. NC: normal control group, AS: asthmatic group, AS + D1: asthmatic group treated with dexamethasone 1 mg/kg/day orally, AS + N20: asthmatic group treated with naringenin 20 mg/kg/day orally, and AS + N40: asthmatic group treated with naringenin 40 mg/kg/day orally (*N* = 6).

The AS group showed a significant increase in SP-D/GAPDH expression compared to the NC group (*P* < 0.001). In the other groups, including AS + D1, AS + N20, and AS + N40, a significant decrease in SP-D/GAPDH expression was observed compared to the NC and AS groups (*P* < 0.001). Also, the AS + N40 group exhibited a significant decrement compared to the AS + N20 group (*P* < 0.001) (Figure 6).

### 3.7. The Effect of Naringenin on the Eosinophils Count in BALF

**Figure 7:**
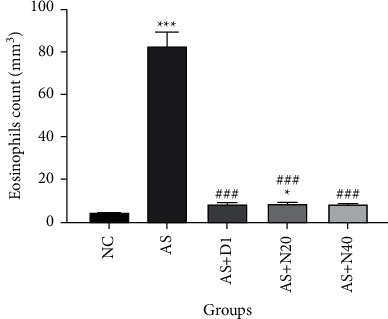
The effect of naringenin on the eosinophil count in BALF. The data are expressed as the mean + SEM. ^*∗∗∗*^*P* < 0.001, ^*∗*^*P* < 0.05, compared to the normal control group. ^###^*P* < 0.001 compared to the asthmatic group. NC: normal control group, AS: asthmatic group, AS + D1: asthmatic group treated with dexamethasone 1 mg/kg/day orally, AS + N20: asthmatic group treated with naringenin 20 mg/kg/day orally, and AS + N40: asthmatic group treated with naringenin 40 mg/kg/day orally, (*N* = 6).

The eosinophil count in the AS and AS + N20 groups showed a significant increase compared to the NC group (*P* < 0.001 and *P* < 0.05, respectively), also treated groups with dexamethasone and naringenin (20 and 40 mg/kg) exhibited a significant decrease compared to the AS group (*P* < 0.001), that confirms the suppression of inflammation in the treated groups (Figure 7).

## 4. Discussion

The results obtained in this study showed that treatment of asthmatic rats with naringenin reduced the levels of inflammatory mediators, such as IL-4 and IL-13 in the lung tissue, controlled oxidative stress via decreasing MDA and increasing GSH levels. Treatment with naringenin also reduced the expression of markers on lung tissue such as UCN and SP-D. Finally, naringenin controlled asthma symptoms in histopathological analysis, so this phytochemical reduced inflammatory cells and airway wall thickness in the histopathological analysis of the lungs.

The Th2 hypothesis, first proposed by Musman in 1982, states that asthma results from a relative increment in Th2 response accompanied by a decrement in Th1 response, resulting in the increment of Th2 products (such as IL-4, IL-5, and IL-13) and a corresponding decrement of Th1 products (such as interferon gamma (IFN-*γ*) and IL-12) [41]. Studies have also shown that the progression of asthma is associated with an increment in the number of eosinophils in lung tissue [2, 4]. The data from our study exhibited that naringenin can effectively improve allergic asthma by reducing the levels of IL-4 and IL-13 in the lung tissue and reducing the count of eosinophils in BALF, which indicates the anti-inflammatory properties of this phytochemical on the pulmonary system.

SP-D and UCN are important genes involved in diseases associated with inflammation. SP-D binds to allergens and pathogens and plays an essential role in reducing eosinophilic inflammation in asthma [42]. Studies have shown that SP-D reduces IL-2 production due to suppression of the proliferation of T-cells [43]. Also, SP-D inhibited the secretion of histamine induced by allergens [44]. Studies confirmed that the expression of SP-D was increased in human and animal models of asthma [45–47]. The data from our study indicated that treatment with naringenin significantly reduced the expression of SP-D, which reduces the symptoms of inflammation in the lung tissue. The results of a study show that intranasal use of SP-D reduced inflammatory symptoms and allergies in mice and also reduced IL-2, IL-4, IL-5, and eosinophil levels [48]. UCN is a proinflammatory factor related to inflammatory conditions that its expression increased in asthmatic conditions due to increasing activity of mast cells and lymphocytes. Studies have shown that UCN has immunosuppressive activity; therefore, it is one of the suitable indicators to evaluate the status of inflammatory diseases [43, 49–51]. It was shown in a study that UCN induced apoptosis in macrophages, thereby controlling inflammation [52]. In another study in a murine model of Crohn's disease, UCN could control inflammatory diseases by inhibiting a wide range of inflammatory cytokines such as TNF-*α*, IFN‐*γ*, IL-6, IL-1*α*, IL-1*β*, IL-12, IL-18, IL-17, and IL-15 [53]. Another study showed that UCN could control neuroinflammation in rats by inhibiting TNF-*α*, IL-6, and IL-1*β* [54]. In the present study, the expression of UCN in lung tissue was significantly reduced by naringenin consumption, which is consistent with previous studies.

Naringenin has been introduced as an immunomodulator phytochemical in several studies [33]. Previous studies have shown that naringenin is known as a phytochemical with strong anti-inflammatory properties. For example, naringenin has been shown to reduce the expression of TNF-*α* and IL-6 in macrophages in the mucus of colon tissue, thereby controlling inflammation [55]. In another study, hat treatment with naringenin reduced TNF-*α*, IL-6, and IL-1*β* expression in hepatocytes by inhibiting the NF-*κ*B pathway [56].

In asthma, on the one hand, the production of a variety of oxygen free radicals increases and the antioxidant level decreases. On the other hand, the concentration of natural antioxidants such as GSH, glutathione peroxidase, superoxide dismutase, and vitamins C and E in the blood, plasma fluid, and BALF of asthmatic patients was reduced. Increased oxygen free radicals, increased peroxidation of proteins, lipids, and nucleic acids, and elevated secretion and permeability of blood vessels were also reported. In addition, oxygen-free radicals initiate the activation of proinflammatory mediators, thereby exacerbating allergic inflammation [57, 58]. The data from our study displayed that naringenin modulates the antioxidant system by decreasing MDA levels and increasing GSH levels in the lung tissue. The antioxidant properties of naringenin have been proven in the treatment of neurodegenerative diseases associated with oxidative stress, such as Parkinson's disease and memory dysfunction induced by diabetes [22, 23]. In other studies performed on ethanol and cadmium-induced oxidative damage, it was shown that naringenin could reduce oxidative damage to the liver by affecting the levels of antioxidant enzymes such as catalase (CAT), GSH, superoxide dismutase (SOD), and glutathione peroxidase (GPx) [24, 59]. Treatment with naringenin also reduces the oxidative stress and DNA damage induced by cisplatin in rats' liver and kidney tissue [60].

## 5. Conclusion

Naringenin dramatically improved ovalbumin-induced allergic asthma through its specific antioxidant and anti-inflammatory characteristics. The therapeutic effects of naringenin in 40 mg/kg were more significant compared to 20 mg/kg.

## Data Availability

The data that support the findings of this study are available from the corresponding author on reasonable request.

## References

[1] Lambrecht B. N., Hammad H. (2012). The airway epithelium in asthma. *Nature Medicine*.

[2] Martin J. G., Tamaoka M. (2006). Rat models of asthma and chronic obstructive lung disease. *Pulmonary Pharmacology & Therapeutics*.

[3] Mittal M., Siddiqui M. R., Tran K., Reddy S. P., Malik A. B. (2014). Reactive oxygen species in inflammation and tissue injury. *Antioxidants and Redox Signaling*.

[4] Scheerens J., van Gessel S. B., Nijkamp F. P., Folkerts G. (2002). Eotaxin protein levels and airway pathology in a mouse model for allergic asthma. *European Journal of Pharmacology*.

[5] Nadeem A., Chhabra S. K., Masood A., Raj H. G. (2003). Increased oxidative stress and altered levels of anti-oxidants in asthma. *Journal of Allergy and Clinical Immunology*.

[6] MacPherson J. C., Comhair S. A., Erzurum S. C. (2001). Eosinophils are a major source of nitric oxide-derived oxidants in severe asthma: characterization of pathways available to eosinophils for generating reactive nitrogen species. *Journal of Immunology*.

[7] Barnes P. J. (1990). Reactive oxygen species and airway inflammation. *Free Radical Biology and Medicine*.

[8] Comhair S. A., Xu W., Ghosh S. (2005). Superoxide dismutase inactivation in pathophysiology of asthmatic airway remodeling and reactivity. *American Journal of Pathology*.

[9] Rahman I., Biswas S. K., Kode A. (2006). Oxidant and anti-oxidant balance in the airways and airway diseases. *European Journal of Pharmacology*.

[10] Barnes P. J., Drazen J. M. (2000). Pathophysiology of asthma. *Asthma and COPD*.

[11] Becker B. (1964). The side effects of corticosteroids. *Investigative Ophthalmology & Visual Science*.

[12] Gandhi G. R., Neta M. T. S. L., Sathiyabama R. G. (2018). Flavonoids as Th1/Th2 cytokines immunomodulators: a systematic review of studies on animal models. *Phytomedicine*.

[13] Park H. S., Kim S. R., Kim J. O., Lee Y. C. (2010). The roles of phytochemicals in bronchial asthma. *Molecules*.

[14] Zhu S., Wang H., Zhang J. (2019). Antiasthmatic activity of quercetin glycosides in neonatal asthmatic rats. *3 Biotech*.

[15] Naeini F., Namkhah Z., Ostadrahimi A., Tutunchi H., Hosseinzadeh-Attar M. J. (2021). A comprehensive systematic review of the effects of naringenin, a citrus-derived flavonoid, on risk factors for nonalcoholic fatty liver disease. *Advances in Nutrition*.

[16] Francis A., Shetty T., Bhattacharya R. (1989). Modulating effect of plant flavonoids on the mutagenicity of N-methyl-N′-nitro-N-nitrosoguanidine. *Carcinogenesis*.

[17] Lee C.-H., Jeong T.-S., Choi Y.-K. (2001). Anti-atherogenic effect of citrus flavonoids, naringin and naringenin, associated with hepatic ACAT and aortic VCAM-1 and MCP-1 in high cholesterol-fed rabbits. *Biochemical and Biophysical Research Communications*.

[18] Salehi B., Fokou P. V. T., Sharifi-Rad M. (2019). The therapeutic potential of naringenin: a review of clinical trials. *Pharmaceuticals*.

[19] Ortiz‐Andrade R., Sánchez‐Salgado J., Navarrete‐Vázquez G. (2008). Antidiabetic and toxicological evaluations of naringenin in normoglycaemic and NIDDM rat models and its implications on extra‐pancreatic glucose regulation. *Diabetes, Obesity and Metabolism*.

[20] Raso G. M., Meli R., Di Carlo G., Pacilio M., Di Carlo R. (2001). Inhibition of inducible nitric oxide synthase and cyclooxygenase-2 expression by flavonoids in macrophage J774A. 1. *Life Sciences*.

[21] Shiratori K., Ohgami K., Ilieva I. (2005). The effects of naringin and naringenin on endotoxin-induced uveitis in rats. *Journal of Ocular Pharmacology & Therapeutics*.

[22] Md S., Alhakamy N. A., Aldawsari H. M., Asfour H. Z. (2019). Neuroprotective and anti-oxidant effect of naringenin-loaded nanoparticles for nose-to-brain delivery. *Brain Sciences*.

[23] Rahigude A., Bhutada P., Kaulaskar S., Aswar M., Otari K. (2012). Participation of anti-oxidant and cholinergic system in protective effect of naringenin against type-2 diabetes-induced memory dysfunction in rats. *Neuroscience*.

[24] Renugadevi J., Prabu S. M. (2010). Cadmium-induced hepatotoxicity in rats and the protective effect of naringenin. *Experimental and Toxicologic Pathology*.

[25] Wang J., Yang Z., Lin L., Zhao Z., Liu Z., Liu X. (2012). Protective effect of naringenin against lead-induced oxidative stress in rats. *Biological Trace Element Research*.

[26] Alberca R. W., Teixeira F. M. E., Beserra D. R. (2020). Perspective: the potential effects of naringenin in COVID-19. *Frontiers in Immunology*.

[27] Chin L. H., Hon C. M., Chellappan D. K. (2020). Molecular mechanisms of action of naringenin in chronic airway diseases. *European Journal of Pharmacology*.

[28] Tutunchi H., Naeini F., Ostadrahimi A., Hosseinzadeh‐Attar M. J. (2020). Naringenin, a flavanone with antiviral and anti‐inflammatory effects: a promising treatment strategy against COVID‐19. *Phytotherapy Research*.

[29] Shi Y., Dai J., Liu H. (2009). Naringenin inhibits allergen-induced airway inflammation and airway responsiveness and inhibits NF-*κ*B activity in a murine model of asthma. *Canadian Journal of Physiology and Pharmacology*.

[30] Iwamura C., Shinoda K., Yoshimura M., Watanabe Y., Obata A., Nakayama T. (2010). Naringenin chalcone suppresses allergic asthma by inhibiting the type-2 function of CD4 T cells. *Allergology International*.

[31] Rothenberg M. E., Hogan S. P. (2006). The eosinophil. *Annual Review of Immunology*.

[32] Moon P.-D., Choi I.-H., Kim H.-M. (2011). Naringenin suppresses the production of thymic stromal lymphopoietin through the blockade of RIP2 and caspase-1 signal cascade in mast cells. *European Journal of Pharmacology*.

[33] Zeng W., Jin L., Zhang F., Zhang C., Liang W. (2018). Naringenin as a potential immunomodulator in therapeutics. *Pharmacological Research*.

[34] Ezz-Eldin Y. M., Aboseif A. A., Khalaf M. M. (2020). Potential anti-inflammatory and immunomodulatory effects of carvacrol against ovalbumin-induced asthma in rats. *Life Sciences*.

[35] Wang J., Wei T., Gao J., He H., Chang X., Yan T. (2015). Effects of Naringenin on inflammation in complete freund’s adjuvant-induced arthritis by regulating Bax/Bcl-2 balance. *Inflammation*.

[36] Moura C. T. M., Bezerra F. C., De Moraes I. M., Magalhães P. J. C., Capaz F. R. (2015). Increased responsiveness to 5‐hydroxytryptamine after antigenic challenge is inhibited by nifedipine and niflumic acid in rat trachea in vitro. *Clinical and Experimental Pharmacology and Physiology*.

[37] Yang E. J., Lee J.-S., Song B. B., Yun C.-Y., Kim D.-H., Kim I. S. (2011). Anti-inflammatory effects of ethanolic extract from Lagerstroemia indica on airway inflammation in mice. *Journal of Ethnopharmacology*.

[38] Sedaghat K., Yousefian Z., Vafaei A. A. (2019). Mesolimbic dopamine system and its modulation by vitamin D in a chronic mild stress model of depression in the rat. *Behavioural Brain Research*.

[39] Liu Y. L., Matsuzaki T., Nakazawa T. (2007). Expression of aquaporin 3 (AQP3) in normal and neoplastic lung tissues. *Human Pathology*.

[40] Wied G. L., Bartels P. H., Bibbo M., Dytch H. E. (1989). Image analysis in quantitative cytopathology and histopathology. *Human Pathology*.

[41] von Hertzen L. C., Haahtela T. (1999). Could the risk of asthma and atopy be reduced by a vaccine that induces a strong T-helper type 1 response?. *American Journal of Respiratory Cell and Molecular Biology*.

[42] Hohlfeld J. M., Erpenbeck V. J., Krug N. (2003). Surfactant proteins SP-A and SP-D as modulators of the allergic inflammation in asthma. *Pathobiology : Journal of Immunopathology, Molecular and Cellular Biology*.

[43] Borron P. J., Crouch E. C., Lewis J. F., Wright J. R., Possmayer F., Fraher L. J. (1998). Recombinant rat surfactant-associated protein D inhibits human T lymphocyte proliferation and IL-2 production. *Journal of Immunology*.

[44] Wang J.-Y., Shieh C.-C., You P.-F., Lei H.-Y., Reid K. B. (1998). Inhibitory effect of pulmonary surfactant proteins A and D on allergen-induced lymphocyte proliferation and histamine release in children with asthma. *American Journal of Respiratory and Critical Care Medicine*.

[45] Cheng G., Ueda T., Numao T. (2000). Increased levels of surfactant protein A and D in bronchoalveolar lavage fluids in patients with bronchial asthma. *European Respiratory Journal*.

[46] Haley K. J., Ciota A., Contreras J. P., Boothby M. R., Perkins D. L., Finn P. W. (2002). Alterations in lung collectins in an adaptive allergic immune response. *American Journal of Physiology - Lung Cellular and Molecular Physiology*.

[47] Kasper M., Sims G., Koslowski R. (2002). Increased surfactant protein D in rat airway goblet and clara cells during ovalbumin‐induced allergic airway inflammation. *Clinical & Experimental Allergy*.

[48] Singh M., Madan T., Waters P., Parida S. K., Sarma P. U., Kishore U. (2003). Protective effects of a recombinant fragment of human surfactant protein D in a murine model of pulmonary hypersensitivity induced by dust mite allergens. *Immunology Letters*.

[49] Karalis K., Sano H., Redwine J., Listwak S., Wilder R. L., Chrousos G. P. (1991). Autocrine or paracrine inflammatory actions of corticotropin-releasing hormone in vivo. *Science*.

[50] Schulte H., Bamberger C., Elsen H., Herrmann G., Bamberger A., Barth J. (1994). Systemic interleukin‐1 *α* and interleukin‐2 secretion in response to acute stress and to corticotropin‐releasing hormone in humans. *European Journal of Clinical Investigation*.

[51] Wu Y., Zhou H., Xu Y., Li S. (2006). Enhanced expression of urocortin in lung tissues of rats with allergic asthma. *Biochemical and Biophysical Research Communications*.

[52] Tsatsanis C., Androulidaki A., Dermitzaki E. (2005). Urocortin 1 and Urocortin 2 induce macrophage apoptosis via CRFR2. *FEBS Letters*.

[53] Gonzalez-Rey E., Fernandez-Martin A., Chorny A., Delgado M. (2006). Therapeutic effect of urocortin and adrenomedullin in a murine model of Crohn’s disease. *Gut*.

[54] Liew H.-K., Pang C.-Y., Hsu C.-W. (2012). Systemic administration of urocortin after intracerebral hemorrhage reduces neurological deficits and neuroinflammation in rats. *Journal of Neuroinflammation*.

[55] Dou W., Zhang J., Sun A. (2013). Protective effect of naringenin against experimental colitis via suppression of toll-like receptor 4/NF-*κ*B signalling. *British Journal of Nutrition*.

[56] Chtourou Y., Fetoui H., Jemai R., Slima A. B., Makni M., Gdoura R. (2015). Naringenin reduces cholesterol-induced hepatic inflammation in rats by modulating matrix metalloproteinases-2, 9 via inhibition of nuclear factor *κ*B pathway. *European Journal of Pharmacology*.

[57] Charavaryamath C., Janardhan K. S., Townsend H. G., Willson P., Singh B. (2005). Multiple exposures to swine barn air induce lung inflammation and airway hyper-responsiveness. *Respiratory Research*.

[58] Murdoch J. R., Lloyd C. M. (2010). Chronic inflammation and asthma. *Mutation Research: Fundamental and Molecular Mechanisms of Mutagenesis*.

[59] Jayaraman J., Veerappan M., Namasivayam N. (2009). Potential beneficial effect of naringenin on lipid peroxidation and anti-oxidant status in rats with ethanol-induced hepatotoxicity. *Journal of Pharmacy and Pharmacology*.

[60] Koyuncu I., Kocyigit A., Gonel A., Arslan E., Durgun M. (2017). The protective effect of naringenin-oxime on cisplatin-induced toxicity in rats. *Biochemistry research international*.

